# Childbirth experiences and satisfaction with birth among women in selected Nigerian healthcare facilities during COVID-19 pandemic: a mixed method

**DOI:** 10.11604/pamj.2024.47.217.38478

**Published:** 2024-04-29

**Authors:** Chikaodili Ndidiamaka Ihudiebube-Splendor, Victoria Uchechi Enwereji-Emeka, Paulina Chigwara Chikeme, Nneka Edith Ubochi, Ngozi Joy Omotola

**Affiliations:** 1 Department of Midwifery, Child Health Nursing, African Centre of Excellence for Public Health and Toxicological Research, University of Port Harcourt, Port Harcourt, Nigeria,; 2 Department of Nursing Sciences, University of Nigeria Nsukka, Enugu Campus, Enugu, Nigeria

**Keywords:** Childbirth experiences, birth satisfaction, social support, COVID-19

## Abstract

**Introduction:**

childbirth experiences are women´s personal feelings and interpretations of birth processes, which could be difficult to describe and explain. The outbreak of Coronavirus disease (COVID-19) instilled tension and worries in all Nigerian citizens and could also affect the birth experiences and satisfaction of women. Thus, this study explored the experiences of childbirth and satisfaction with birth among women in selected Nigerian healthcare facilities during COVID-19 pandemic.

**Methods:**

the study adopted a concurrent triangulation mixed method design, which utilized an in-depth interview and questionnaire to obtain different but complementary data. Sample sizes of 304 and 15 women were recruited for quantitative and qualitative data, respectively. Analysis was done using descriptive statistics and thematic content analysis.

**Results:**

the majority of the participants perceived childbirth to be labor and delivery (3.66 ± 3.16); participants were mostly satisfied with reception received from staff (2.35 ± 2.29) and respecting their privacy (2.04 ± 1.52). Five (5) themes and 18 subthemes emerged from qualitative data. The themes were: understanding of childbirth, satisfaction with care, hospital experiences, unique experiences during birth, and social support.

**Conclusion:**

women had more positive and less negative but unique childbirth experiences. The majority expressed satisfaction within the care given by qualified and competent health workers, despite the challenges posed by COVID-19 pandemic. The provision of physical and emotional support by intimate partners, midwives´ and family members during delivery had a significant influence on maternal satisfaction with the entire birth experience.

## Introduction

Childbirth is a multifaceted process with physical and psychosociocultural dimensions [1] which are difficult to describe and explain. Birth experiences are mother´s personal feelings and interpretations of birth processes which could be positive, improving mother´s wellbeing and maternal-child bonding, or negative, resulting in a condition of emotional distress such as postnatal depression [2]. Various variables are involved in the assessment of childbirth experiences, including expectations of labor, midwives support, and use of invasive methods such as episiotomies, among others [3]. Though a lot is known about the clinical management of labor and childbirth, little attention is given to what ought to be done to make mothers feel satisfied, and positive about the experience of birth [4]. Women´s personal stories show the importance of birth in their lives. Hence, understanding maternal perception of care and satisfaction with services is important, as perceived quality is a key determinant of service utilization [5]. Satisfaction of expectant mothers´ psycho-socio-cultural needs is an important criterion for assessing quality care which is readily provided by the midwives [5-7]. It has also been reported that health facilities at different care levels globally have been encountering difficulties in ensuring quality care and client´s satisfaction. Conversely, assessing the quality of childbirth care from client´s viewpoint is a crucial aspect of quality care and how the skilled birth attendants deliver their services during birth period can result in positive or negative experiences [8].

However, the COVID-19 (Coronavirus disease) pandemic inflicted the whole nations of the world with a great shock, thereby overwhelming the global healthcare systems. Between March and June 2020, Nigeria recorded a sharp rise in the total number of confirmed cases. The Nigeria Centre for Disease Control (NCDC) on 7^th^ June announced 12,486 confirmed cases in the country: 260 new cases of COVID-19 with Abia State recording the highest daily figure with 67 infections, more than four times its initial 16 infections since February 2020 [9]. Anecdotally, this has instilled tension and worries in all citizens and could also affect the birth experiences and satisfaction of women in the maternity unit. With COVID-19 emergence in Nigeria, a greater number of pregnant women did not experience a fair treatment going to the health facilities for their antenatal care and delivery [10]. Although the severity of COVID-19 infection in pregnant women is still being determined, pregnant women are considered a high-risk population [11,12]. Countries struggling with the pandemic may need to divert important resources, including skilled birth attendants, from daily service delivery to response efforts. More so, expectant mothers may experience challenges accessing services due to transport alterations and lockdown guidelines or be unwilling to visit the healthcare facilities because of fear of infection. However, there is dearth of documented studies on birth experiences and satisfaction among women during the COVID-19 pandemic in Nigeria. Thus, the study was set to explore the birth experiences and satisfaction with birth among women in selected healthcare facilities in Umuahia Abia State, Southeast Nigeria during the peak period of COVID-19.

## Methods

**Study design and participants:** the study adopted a concurrent triangulation mixed method research design to obtain different but complementary data among women that delivered their babies and attending Infant welfare clinic in the selected healthcare facilities (Federal Medical Centre (FMC) Umuahia and Abia State specialist Diagnostic Centre Umuahia) between March and December 2020. These health facilities provide specialized and comprehensive healthcare services, including maternity care. In addition, they were designated COVID-19 Centres during the period of the pandemic to provide treatment to patients with such diagnosis. For the quantitative study, a sample size of 304 was calculated from a total population of, 1268 using Taro Yamene´s formular


$$n=\frac{N}{1+N{\left(e\right)}^{2}}$$

Where n= sample size; N= population size; e= level of precision or sampling error, which is ± 5% and participants who met the following inclusion criteria: aged between 20 and 49 years, had no coexisting medical condition and consented to participate in the study were purposively recruited. For the qualitative aspect, fifteen (15) women were recruited through snowballing. Cormack suggests that qualitative researchers use a small selective sample due to the detailed nature of their study and data analysis required [13,14].

**Data collection:** data were collected using validated researchers-developed questionnaire for quantitative data and interview guide for qualitative data. The questionnaire elicited information on participants´ demographics and their perceptions and satisfactions towards childbirth, while the interview guide explored the meaning of childbirth, birth experiences and women´s satisfaction with birth during the period of COVID-19 pandemic. The quantitative instrument was pretested using a group of women with similar features in General Hospital Aba; a test-retest method was applied, and data obtained were analyzed using Cronbach Alpha test, which yielded a reliability coefficient of 0.75. For the qualitative data collection, the principles of rigor and trustworthiness were achieved through credibility, transferability, dependability and conformability according to qualitative researchers [15-17]. All interviews were audio-recorded with the permission of participants to ascertain an accurate account of the interview, which were replayed for analytic purposes. The interview continued until the fifteenth participant, when no new information was obtained. All the audio-recorded interviews and discussions were transcribed verbatim and the ones done in Ibo language were translated to English. Anonymous quotes were used to illustrate the fact.

**Data analysis:** quantitative data were subjected to descriptive statistics of frequencies, percentages, mean and standard deviation and analysis done with the aid of Statistical Package for Social Sciences (SPSS) software, (version 22) while the qualitative aspects were analyzed inductively using thematic content analysis described by Graneheim and Lundman [18]. Applying three levels of coding, firstly, the researchers listened to the recorded data over and again, read and reread the transcripts and field notes, examined data line by line and made codes from the language of the participants. Second level coding involves comparing the coded data, clustering and creating categories/labels from them according to research questions. In third level coding, a central theme was derived from each of the categories by aggregating similar codes; subthemes were also extracted. The themes were then used to describe the phenomenon and answer the research questions.

**Ethical approval:** for the study was obtained from Ethics and Research Committees of Federal Medical Centre Umuahia (FMC/QEH/G.596/Vol.10/503) and University of Port Harcourt (UPH/CEREMAD/REC/MM75/072). Administrative permits were obtained from the Unit heads, while written informed consents were obtained from the participants.

## Results

Results from quantitative data revealed that out of 304 copies of questionnaire distributed, only 280 were returned giving a 92% retrieval rate. The mean and standard deviation age is 25.87±3.18; majority 195 (69.64%) had tertiary education; predominantly Christians 270 (96.43%); Primiparous 150 (53.57%); and majority had normal vaginal delivery 180 (64.29%). Based on the facility, 200 (71.43%) were from FMC Umuahia while 80 (28.57%) were from Abia State specialist Diagnostic Centre Umuahia (Table 1). Majority of the participants perceived childbirth to be labor and delivery (3.66 ± 3.16) while others perceived it to be the process of delivering a baby, placenta, and membrane (3.50 ± 3.06) and the act of having a baby (3.50 ± 3.01) (Table 2). Majority of participants were mostly satisfied with reception received from staff (2.35 ± 2.29) and respecting the privacy of patients (2.04 ± 1.52) (Table 3). Results of qualitative data showed that 15 women were interviewed about their childbirth experiences and satisfaction. Their socio- demographic variables revealed that 10 (66.67%) were from FMC Umuahia, 5 (33.33%) from Abia State specialist centre. Majority were aged between 25 and 35 years; had normal deliveries 11 (73.33%) and had delivered 2-4 times 9 (60.00%), all were married and Christians, had formal education (Table 4). Five (5) themes and 18 subthemes emerged from data analysis. The themes were: understanding of childbirth, satisfaction with care, hospital experiences, unique experiences during birth, and social support (Table 5).

**Table 1:** sociodemographic characteristics of the participants (n=280)

Variable	Frequency	Percentage
**Health facility**		
Federal medical centre	200	71.43
Abia State Specialist Centre	80	28.57
**Age (years)**		
<25	104	37.15
25 - 30	150	53.57
31 - 35	20	7.14
Above 35	6	2.14
M±SD age =25.87±3.18		
**Highest educational level**		
No formal education	0	0.00
Primary	0	0.00
Secondary	85	30.36
Tertiary	195	69.64
**Marital status**		
Married	260	92.86
Single	20	7.14
Divorced/widowed	0	0.00
**Occupational status**		
Student	4	1.43
Housewife	8	2.86
Business	200	71.43
Civil servant	68	24.29
**Religion**		
Christianity	270	96.43
Islam	0	0.00
No religion	10	3.57
**Socio-economic status**		
Earns N10000 - N50000 monthly	198	70.71
Earns N50000 - N100000 monthly	62	22.15
Earns above N100000 monthly	20	7.14
**Parity**		
Has given birth once	150	53.57
Has given birth 2-4 times	120	42.86
Has given birth 5 times and more	10	3.57
**Type of delivery**		
Normal	180	64.29
Instrumental	20	7.14
Caesarean section	80	28.57

**Table 2:** perception about childbirth (n=280)

Statements	Strongly agree	Agree	Disagree	Strongly disagree	Mean±SD
The act of having a baby	150 (53.57)	120 (42.86)	10 (3.57)	0 (0.00)	3.50±3.01
Labour and delivery	188 (67.14)	90 (32.14)	1 (0.36)	1 (0.36)	3.66±3.16
Process of delivering a baby, placenta, and membrane	180 (64.29)	70 (25.00)	20 (7.14)	10 (3.57)	3.50±3.06

**Table 3:** birth satisfaction of women during childbirth (n=280)

Statements how satisfied with	Very dissatisfied	Dissatisfied	Neutral	Satisfied	Very satisfied	Mean±SD
Services received	5 (1.79)	10 (3.57)	5 (1.79)	80 (28.57)	180 (64.29)	1.50±1.21
Cleanliness of the facility	2 (0.70)	6 (2.14)	2 (0.70)	260 (92.86)	10 (3.57)	1.39±1.02
Reception from staff	1 (0.36)	1 (0.36)	8 (2.86)	70 (25.00)	200 (71.43)	2.35±2.29
Time taken to attend to patients	40 (14.29)	38 (13.57)	2 (0.70)	100 (35.71)	100 (35.71)	1.33±0.89
Knowledge and competence of health workers	12 (4.29)	15 (5.36)	3 (1.07)	100 (35.71)	150 (53.57)	1.71±1.51
Respecting privacy of patients	3 (1.07)	5 (1.79)	2 (0.70)	80 (28.57)	190 (67.86)	2.04±1.52
Responses from health staff	10 (3.57)	20 (7.14)	10 (3.57)	90 (32.14)	150 (53.57)	1.75±1.56
Guidance given during labor	10 (3.57)	5 (1.79)	5 (1.79)	60 (21.43)	200 (71.43)	1.45±1.21

**Table 4:** socio-demographic characteristics of participants in qualitative study (n=15)

Variables	Frequency (%)
**Health facility**	
Federal medical centre	10(66.67)
Abia State specialist centre	5(33.33)
**Age (Years)**	
< 25	4(26.67)
25-30	8(53.33)
31-35	3(20.00)
>35 0	0(0.00)
**Marital status**	
Married	15(100)
Single	0(0.00)
Divorced /widowed	0(0.00)
**Religion**	
Christianity	15(100)
Islam	0(0.00)
No religion	0(0.00)
**Highest educational level**	
No formal education	0(0.00)
Primary	4(26.67)
Secondary	5(33.33)
Tertiary	6(40.00)
**Occupation**	
Students	4 (26.66)
Housewife	3(20.00
Business	4(26.66)
Civil servant	4(26.66)
**Parity**	
Has given birth once	4(26.67)
2-4 times	9(60.00)
More than 4 times	2(13.33)
**Type of delivery**	
Normal	11(73.33)
Others specify (CS)	4(26.67)

**Table 5:** core themes and subthemes derived through thematic content analysis

Main themes	Subthemes
Understanding of childbirth	Power of God in the events of birth
	Self-strength in the event of birth
	Courage to push when the baby is coming out
Hospital experiences	Delay in accessing care due to lockdown protocols
	Physical environment
	Hospital protocols
	Disruption to quality care
Unique experiences during childbirth	Natural experience
	Unremarkable feelings
	Troublesome experience
	Interventions undertaken with consent
	Anxiety/fear of being infection by Covid-19
Satisfaction with care	Competency of staff
	Timely response to clients
	Midwife’s attitude towards care delivery
Social support	Intimate partner experience and advice
	Midwife’s support
	Family member’s support

**Theme 1: understanding of childbirth** the women reported that childbirth is determined by the power of God together with self-strength/courage.

**Power of GOD in the events of birth:** the power of God in the events of birth was held in high esteem. One woman expressed; “*Without God´s abundant grace, a woman cannot deliver safely especially during this critical corona-virus era (participant 2 from FMC).”* A 34-year-old Para 2 mother exclaimed, “*Before my date of delivery reached, my church women organized a day fasting and prayer for me. I was considering delivering at the health center close to my house due to the lockdown wahala but the spirit of God ministered to me to go to Abia specialist… (participant 2 from Abia specialist).”*

**Self-strength in the events of birth:** a mother of one reported, “*I was told by a mother that the midwives beat women in labor if they don´t have strength to push. As a first-time mother, I believed in my strength …, my delivery was safe (participant 1 from FMC).”*

**Courage to push when the baby is coming out:** “*Hmmmm! my sister during the time of pushing the baby, the pains that comes is too intense if you don´t have courage, you can even give up, and your baby will suffer...” (participant 1 from Abia specialist)”*.

### Theme 2: satisfaction with care

**Physical environment (structure):** R.U stated, “*I am comfortable with the way the maternity ward is built in sections. Beds are comfortable, each woman has separate corner, … what I didn´t like was too much mosquito bite, at night I couldn´t sleep as I was busy chasing mosquitoes from my twin babies.” (participant 10 from FMC)* Mrs J. states: “*I like the environment inside the ward, it is always neat, but outside environment was bushy... Even when there was no electricity ‘ha ga-etinyere anyi igweoku´ meaning ‘they will switch on generator to give us light (participant 5 from Abia specialist).”*

**Qualified and competent staff (structure):** excerpts from some mothers. Mrs S´s responses were: “*Nwannem Nwanyi” meaning ‘my dear Sister´. FMC Umuahia has experts and professionals that are competent in their services especially during emergency. I didn´t have in mind to do operation but because of low progress in labor I was prepared and sectioned within minutes (participant 3 from FMC).”* While Mrs G states: *“I heard that Government hospitals employ only those who read book that concern women well. That is why I registered here, ... I have had three miscarriages because I never went to government hospital before, but to a woman that born children in my village. I eventually saw the difference this time; you can see my bouncing baby (participant 3 from Abia specialist).”*

**Adequacy of staff (structure):** “*Some shift, like morning operates with adequate number of staff but evening and night shifts operates with few staff. When I was in labor, about three other women were also in labor, and we were screaming Nurse o, Nurse o, Nurse lee! ´Nsi na-akpa mu oo´ meaning ‘Nurse I feel like emptying my bowel´. The nurse didn´t know who to attend to first but managed to attend to us one after another (participant 5 from FMC)”*.

**Timely response to clients by the midwives (process):** Mrs N. in her response states: “*I asked the midwives whether the signs they saw showed that myself and baby were in good condition, they answered all my questions… However, I would like to recommend a relative to deliver there´ (participant 4 from Abia specialist)”*. Another mother expressed dissatisfaction stating thus: “*In fact, a big hospital like this should have enough midwives during every shift. When my labor started in the morning, many midwives were on duty … when I was about to deliver my baby, very few were on duty and also busy, you will shout and shout for them to come and check you…, until the baby starts coming out by that time, they will know that you are serious” (participant 7 from FMC)*.

**Midwives attitude towards care delivery:** K.C a para 2 mother stated, “*I preferred the attitude of the nurses and midwives to doctors the time I delivered. They treated me with respect and provides protection during every examination. The doctors had “bad mouth” and would ignore you when you call them, but the midwives gave me hope when I was pushing; (participant 9 from FMC).”* Mrs G narrated: *“If I should rate my level of satisfaction, I will score them 50% because with all the qualified health staff there, … I was also dissatisfied with their attitude of delay in making my bills and the stress of queueing up at the immunization clinic…, instead of the nurses to come to the ward and immunize the babies (participant 3 from Abia specialist).”*

**Delay in accessing care due to lockdown protocols:** Mrs Z stated *“The experience from home to the hospital was so frustrating due to stay-at-home rules. The security agents don´t even want to know .... so many roadblocks, and you must stop at each check point to answer questions why you are on the road (participant 1 from FMC).”* Another mother affirmed. *“I nearly delivered on the road, if not that I was shouting at every check point. In fact! The experience was very terrible´´ (participant 5 from Abia specialist)*

**Hospital protocols:** a woman narrated, *“I passed through rigorous processes with my mum who accompanied me, despite my labor pains. Immediately we stepped into the hospital gate, the security men directed us to wash our hands and sanitize…, in the labor ward, the process was also repeated… In fact, I became so tired of the whole process” (participant 7 from FMC)*. Another mother gave her own story. *“The hospital procedures were so strict. Wearing of masks was mandatory even throughout labor, delivery and after which created a challenge for women (participant 1 from Abia specialist)*.

**Disruption of quality care:** mother Z stated, *“The COVID-19 protocol affected the care given by the midwives. During my previous delivery, the midwives were calm in giving their care and they observed me very well, but this time, they looked strict and anxious and if you are not with your face mask or sanitizer, they would not attend to you.” (participant 1 from FMC) Yet another mother responded, “I believe that COVID-19 is real and the protocols like wearing of face masks, frequent hand washing, etc did not prevent the midwives from doing their work very well because their aim is to prevent spread of infection” (participant 4 from Abia specialist)*.

**Theme 4: unique experiences during childbirth**

**Natural experiences:** Mrs. U, a mother of twins exclaimed “*Ahhh my Nurse! that COVID-19 period was really in my favor oo… I felt I would deliver my twins on the road but luckily for me, we reached hospital safely. The midwives were so receptive, treated me with respect, one of the midwives rubbed my back but did not allow my husband to come in... I was very happy with the services I received from them till discharge” (participant 10 from FMC)*. Another mother from Abia specialist says, *“I considered my expectations met when my baby was born safely through a normal delivery and both of us were safe” (participant 4 from Abia specialist)*.

**Unremarkable feelings:** Mrs S, a para-4 mother affirmed, *“Although I delivered through surgery, but everything was successful. You can see that my baby and I are in sound health” (participant 3 from FMC)*. Another first-time mother says, *“What mattered most to me was that both my baby and I came out of labor successfully. I don´t care much about the attitude of health workers” (participant 3 from Abia specialist)*.

**Troublesome experiences:** a 32-year-old mother narrated, *“During the time I was having strong contractions, I felt like pooing, I called the midwives and doctors to come and check me, they were busy discussing Chelsea and Man U, ... The moment I shouted that the head of my baby was already out, all of them rushed in and delivered my baby. Minutes after delivery, I started bleeding profusely due to what they called “tear” I nearly passed out at the postnatal ward. God really saved me my sister” (participant 7 from FMC)*. Another mother from Abia specialist affirmed; *“Their delivery room has some equipment but not all are in good condition. For example, I delivered my baby who did not breath well, the machine to suck him was faulty and I was afraid that my baby would die that day until they went to the theatre to bring another machine. I thank God …” (participant 3 from Abia specialist)*.

**Interventions undertaken with consent:** a mother from Abia specialist exclaimed, *“Wowww! The health workers were so nice to me, they gave me a warmth reception... and explained every procedure to me. Indeed, I was treated well.”* Another mother from FMC narrated her own experience as stated: *“Even though the midwives and doctors were very sensitive about observing COVID-19 Protocols, they treated me well by encouraging me, explained every procedure before carrying out…, although I was giving a cut during delivery to make my baby come out easily, the pains were suppressed when I heard my baby crying.”*

### Anxiety/fear of being infected

**Excerpts from some participants:** a 25-year-old Para 1, mother reported, *“I was so anxious during the time of my delivery because I was told during one of my ANC teachings that pregnant women are more vulnerable to corona virus… Much of my worry was that my husband who is a health worker was always in contact with so many people, I was afraid he would be infected and give it to me and my baby meanwhile he didn´t believe that COVID-19 was real and did not take the protocols very serious.” (participant 4 from FMC)*. Another mother expressed, *“Hmmm! The tales about corona itself, the daily news updates both fake and real, the increment in number of confirmed cases and deaths, … embarrassment on the road by tax force operators, all these caused me a lot of tension during pregnancy and childbirth” (participant 5 from FMC)*.

### Theme 5: social support

**Midwife´s support:** a mother expressed thus, *“I was properly supported by the midwives. Despite the protocol of social distancing, I was not isolated, they constantly checked on me and my baby and also counseled me on breastfeeding and care of my baby” (participant 5 from Abia specialist)*. Mrs. N.K expressed, *“I felt so exhausted and discouraged, they neglected me when I requested for back massage during labor pains. What annoyed me so much was that they will tell you they are coming but they never did… visitors were strictly restricted because of COVID” (participant 6 from FMC)*. Intimate partner experience and advice an excerpt from a mother state: *“My happiness was that my husband was around to run errands for me especially taking my baby to immunization clinic for BCG. He also arranged for her mum´s visit and advised me to be patient with hospital protocols...” (participant 2 from Abia specialist)*.

**Family members support:** on the part of support from family members, one mother narrated, *“My mother and sister were not allowed to come into the labor ward. I felt so lonely and frustrated with labor pains,..., they only talked through the window to tell me ‘Ndo, jisieike´ meaning ‘sorry and be strong´. The midwives on duty were only few and busy” (participant 2 from FMC). Yet another mother expressed thus, “My mother and siblings who visited me after delivery were not allowed to enter the ward. The midwives collected what they brought and gave me telling them to come to the house when I am discharged, my baby was not even bathed like before; they only cleaned her up (participant 3 from Abia specialist)*.

## Discussion

The knowledge of what childbirth means is the most important step in handling and managing pregnancy till the day of delivery. The quantitative findings revealed that most mothers understood childbirth to be a process of going through a series of stress, commonly known as ´labor´. Labor stages, thereafter usher in the delivery stage, which involves both delivery of the baby and afterbirths. This is similar to reports of George and Solomon, [19] and Slade *et al*. [20] that explained childbirth as a physiological process of having a baby after passing through different stages. On the other hand, participants from the qualitative study, expressed their understanding of childbirth in three-folds which were, the power of God in the event of birth, self-strength and courage during the time of pushing the baby. To these mothers, having God and trusting in His power according to Biblical injections enable women to have normal and safe deliver. Similar to a report by Furman [21] who opined that labour and birth is seen in the frame of the broader biblical account, inculcating a huge meaning into their individual experiences and keeping their focus off of themselves and onto God. The participants also reported that self-strength/courage enhanced their birth success as they tolerated the painful contractions. According to Fenwick *et al*. belief in oneself and courage has been reported to enhance a positive childbirth experience. In addition, most African cultures would expect women to give birth vaginally and if not, would be considered weakling and not good enough. Such beliefs can also cause loss of self-esteem, resulting in poor adaptation to motherhood [22]. Adequate knowledge of the religious and cultural interpretation of childbirth helps the midwives to provide psychological support to those affected and their families, and also help policymakers to develop interventions to bridge knowledge gaps regarding birth options.

With COVID-19 pandemic, maternal and child health services were greatly influenced in Nigeria. The participants narrated more negative than positive experiences in accessing maternal care due to lockdown and sit-at-home order by the government; transport interruptions, with many check-points and road blocks, thereby making movement from home to hospital very frustrating for women in labor. According to some of the participants, they nearly delivered on their way to the hospital if not that they shouted at every check points to scare the security operatives. The few who were favored were those whose husbands conveyed to the hospital. This finding is in line with the reports of Mollard and Wittmaack, that greater number of pregnant women did not experience a fair treatment going to the health facilities for their antenatal care and delivery, due to lockdown measures [23], and fear of getting infected in the hospital [24]. The study also revealed that the hospitals were strict in patient´s observing COVID-19 protocols as the women were mandated to pass through one entrance for their temperature check, sanitize their hands, wear face mask even while pushing the baby which made them very uncomfortable. This is consistent with the labor and delivery guideline [25] which states that guidelines for maternal care practices should promote the feelings of safety and control and also overall experience of women giving birth in hospitals during any pandemic. Some participants narrated that quality care by midwives were disrupted due to COVID-19 protocols, unlike better care received during their previous pregnancies. They expressed that the midwives were strict in observing COVID-19 protocols, while others who did not believe in the reality of COVID-19 frowned at this attitude. More so, the participants had more positive and less negative and unique experiences. Despite the challenges of lockdown, some were favored by having their husbands around to take them to the hospital when labor started and special care given by midwives throughout labor to safe delivery. Some considered their expectations met when they had a safe birth whether through normal vaginal or caesarean birth. This finding is similar to reports from Uganda on healthcare workers support and intervention outcomes on experiences of childbirth which stated that some participants reported positive experiences on physiologic and emotional support given and negative experiences on improper conversation and care [24]. Another study in the United States found that proper conversation between mothers and healthcare workers at the time of birth had a positive result on the birth experiences [23].

Those who had troublesome experiences associated it with delay in response by the midwives, poor working equipments, and poor access to delivery materials. This agrees with Khresheh´s report that mothers who are not adequately assisted by midwives during childbirth relatively may report negative birth experiences [26]. More so, most participants in the study expressed fear and anxiety due to widespread rumors on increasing number of confirmed cases of COVID-19 in the State, fear of exposing their newborns to COVID-19 infection, facility designated as isolation center, and intimate relationship with some husbands who do not abide to COVID-19 protocols because of their unbelief on existence of COVID-19. This corresponds with the report of Fetters who posited that a worldwide pandemic brings multiple layers of logistical and psychological stress to the already stressful period of new parenthood [27]. The subthemes explored for satisfaction were based on staff competency, timely response to client´s needs, and midwives attitude towards care delivery. Birth satisfaction and perception of quality of care are extremely important but often neglected aspects of healthcare services in Nigeria because there are no measures established for frequent assessment of this aspect of quality of care [28]. It has been documented that mothers´ perception of the quality of care and their satisfaction during childbirth is paramount in determining the status of maternal mortality and morbidity in the country [29]. Overall, very few mothers expressed dissatisfaction with the services provided at the hospitals, whereas majority expressed satisfaction with the care given by qualified and competent health workers. This result is similar to Olarinmoye *et al*. previous work on client satisfaction in Nigeria, which also reported that over 80% of clients were satisfied with services provided in the healthcare facilities [30].

This indicates that Nigerian healthcare workers are doing a great job at their various hospitals, and their efforts should be encouraged by providing adequate resources needed to give standard quality care. Furthermore, other aspects of care that participants expressed their satisfaction were cleanliness of the hospital environment and wards, warmth reception by staff, and respecting their privacy while carrying out any procedure among others. This result is consistent with the study conducted in Ethiopia, which showed that most participants were satisfied with respect and assurance of privacy, response from health staff, and guidance given during labor [31]. However, maintenance of privacy is an important requirement for women during physical examinations and delivery process itself in order to protect and respect their dignity. Previous studies have shown that women reiterated the need to provide adequate privacy during physical examinations and procedures by shielding them from other women or visitors [32-35]. Hence, promoting respectful maternity care is well recognized across the globe as one of the key measures for enhancing utilization and quality maternal care [36]. The experiences of women regarding support ranged from positive to negative and discouraging. Some participants in this study expressed their satisfaction with emotional support and encouragement by the midwives despite maintenance of social distancing. Some were also allowed to take sips of water, change their position during the first stage of labor and counseled on breastfeeding/care of themselves and baby; while some women felt discouraged in such aspects as neglecting their request due to inadequate and busy staff on duty, not providing support like sacral message during contractions, or drinks to prevent exhaustion among others.

The provision of physical and emotional support during delivery has a significant influence on maternal satisfaction with the entire birth experience. When this aspect of care is neglected, it could lead to increased anxiety on expectant mothers. This finding is similar to a study conducted in Jordan and South Africa where it was reported that midwives offered limited emotional support during childbirth [34,37] but had better support from partners, relatives and friends during delivery [34]. Low support from the midwives could be attributed to the limited number of midwives on duty to attend to more than one woman at a time during labor. Mothers who are not adequately assisted by midwives in the course of delivery may report a relatively negative birth experience. The study also revealed the high level of support women received in the postnatal ward for breastfeeding. Most participants were encouraged to breastfeed their newborns. Women expressed satisfaction seeing midwives supporting them to exclusively breastfeed their babies. Dykes [38] in an ethnographic study in England posited the needs of breastfeeding women for informational and practical support as well as for emotional, and esteem needs to be largely met. The study also revealed dissatisfaction with lack of intimate partner relationship/advice and family members support due to COVID-19 restrictions. This is in line with the findings of Mollard and Withmaack that women may feel less supported during the experience of childbirth because of variations in maternity ward practices due to the pandemic [23].

## Conclusion

This study explored childbirth experiences and satisfaction with birth among women in selected Nigerian healthcare facilities during COVID-19 pandemic using a mixed method. Major findings revealed that most women understood childbirth to not only giving birth, butia process of going through series of stress commonly known as ´labor´. Women´s personal stories showed the importance of birth in their lives, both the positive nature of normal birth and negative effects some interventions might have. Hence, most participants had more positive and less negative and unique chidbirth experiences during the pandemic. Very few mothers expressed dissatisfaction with the services provided at the hospitals, whereas the majority expressed satisfaction with the care given by qualified and competent health workers. The experiences of women regarding social supports ranged from positive to negative and discouraging because of variations in maternity ward practices due to the pandemic. Hence, this is an important study that should influence maternal and child health policy at both the district and national levels. Nevertheless, this study had some limitations such as risk of response bias, in which the women might tend to offer socially desirable responses when asked about their birth experiences and satisfaction with birth or exaggerate their fears, especially on rumors surrounding the spread of COVID-19. Also, findings are context-bound to the participants and settings in which recruitment occurred, while generalizability is not a prerequisite of qualitative research, the findings of the study can be verified.

### What is known about this topic

- *Quality maternal childcare service during pandemic is very important to maintain positive experiences and better satisfaction of women during childbirth;*
- *Mobilization of the healthcare systems are very crucial for alleviating pregnant women’s difficulties in situations such as the COVID-19 pandemic;*
- *Continuous supports by skilled birth attendants during labor may improve outcomes for women and newborns, including reduced negative feelings about childbirth experiences*.

### What this study adds

- *Women’s experiences of giving birth will be a pleasant and enjoyable one if they are actively involved;*
- *Pandemic-related changes to maternity care practices have impacted on birthing women's feelings of safety and support in the hospital environment;*
- *Quality maternal childcare that is consistent with women expectations during birth, especially in times of pandemics will result to positive birth experiences for women*.
